# Slowly Digestible Carbohydrate for Balanced Energy: In Vitro and In Vivo Evidence

**DOI:** 10.3390/nu9111230

**Published:** 2017-11-10

**Authors:** Vishnupriya Gourineni, Maria L. Stewart, Rob Skorge, Bernard C. Sekula

**Affiliations:** Global Nutrition R & D, Ingredion Incorporated, 10 Finderne Ave, Bridgewater, NJ 08807, USA; maria.stewart@ingredion.com (M.L.S.), rob.skorge@ingredion.com (R.S.) bernie.sekula@ingredion.com (B.C.S.)

**Keywords:** slowly digestible carbohydrates (SDC), slowly digestible starch (SDS), sustained energy, sustained blood glucose

## Abstract

There is growing interest among consumers in foods for sustained energy management, and an increasing number of ingredients are emerging to address this demand. The SUSTRA™ 2434 slowly digestible carbohydrate is a blend of tapioca flour and corn starch, with the potential to provide balanced energy after a meal. The aim of the study was to characterize this starch’s digestion profile in vitro (modified Englyst assay) and in vivo (intact and cecectomized rooster study), and to determine its effects on available energy, by measuring post-prandial glycemia in healthy adults (*n* = 14), in a randomized, double-blind, placebo-controlled, cross-over study, with two food forms: cold-pressed bar and pudding. The in vitro starch digestion yielded a high slowly digestible fraction (51%) compared to maltodextrin (9%). In the rooster digestibility model, the starch was highly digestible (94%). Consumption of slowly digestible starch (SDS), in an instant pudding or bar, yielded a significantly lower glycemic index compared to a control. At individual time points, the SDS bar and pudding yielded blood glucose levels with significantly lower values at 30–60 min and significantly higher values at 120–240 min, demonstrating a balanced energy release. This is the first study to comprehensively characterize the physiological responses to slowly digestible starch (tapioca and corn blend) in in vitro and in vivo studies.

## 1. Introduction

According to the World Health Organization (WHO), diabetes will be one of the leading causes of deaths by 2030 [1,2]. Elevated post-prandial glycemia may be one of many risk factors contributing to chronic diseases, such as diabetes [3]. Improvement of the blood glucose response to carbohydrate is pivotal for diabetes management. Carbohydrates influence blood glucose and are ranked using a standardized measurement, the glycemic index (GI). In a meta-analysis of 12 randomized trials, low GI diets, compared to high GI diets, were shown to improve the glycemic response by lowering glycated albumin (HbA1c), reaching levels of clinical significance in diabetes [4]. Low GI diets (GI < 55) release glucose at a sustainable rate, thus providing balanced available energy, and health benefits, such as diabetes management and improved heart health [4,5].

Carbohydrates are a commonly consumed staple food and represent 45–55% of daily energy intake in a Western diet [6]. Carbohydrates play an important role as preferred substrates for brain and red blood cells; thus, the Institute of Medicine (IOM) established a recommended dietary allowance (RDA) for carbohydrates, of 130 g/day for adults and children at least 1 year of age. Food starches derived from cereals, legumes, tubers have semi-crystalline granules, which differ in size and shape, amylose and amylopectin content, chain lengths, degree of branching and X-ray diffraction patterns. Earlier studies reported the influence of starch structure on its digestibility [7]. The digestion rate of starch is measured in vitro by the Englyst method, which classifies starch into three fractions: rapidly digestible starch (RDS), slowly digestible starch (SDS) and non-digestible resistant starch (RS) [8].

Some native starches have inherently high SDS content (A-type X-ray diffraction pattern granule), such as in waxy corn starch or RS (B-type X-ray diffraction pattern granule), such as in potato starch [9]. During processing in the presence of heat and moisture, SDS/RS content is replaced by RDS due to changes in the structural order of starch. This leads to increased accessibility to digestive enzymes and rapid release of glucose. This phenomenon is described as starch gelatinization [10]. Starch gelatinization influences SDS content. Cereal products with high SDS contents and limited starch gelatinization, such as breakfast biscuits, have been shown to have a low GI [11,12].

Starch digestibility is associated with GI. Slowly digestible carbohydrates, commonly abbreviated as SDC, are digested steadily, resulting in prolonged glucose release from the lumen of the small intestine into the blood stream, with blunted glycemia and therefore a lower insulin requirement [13]. Because SDCs provide a high amount of available carbohydrate and are typically low in dietary fiber, they can be formulated into products that have a low GI. Low GI carbohydrates, such as SDCs, include certain types of sugars and starch [11]. There are a few commercial SDCs for sustained energy management, including isomaltulose (disaccharide), trehalose (disaccharide), sucromalt (alternan oligosaccharide) and pullulans (maltotriose-based polysaccharide) [14]. The aforementioned SDCs may have application challenges due to their inherent albeit low sweetness and inability to provide textural functionality. Starch-based SDCs have the advantage of allowing easy replacement of RDS with SDS, in bakery, snack and beverage applications.

The investigational material in the present study is a new, commercially available product, the SUSTRA™ 2434 slowly digestible carbohydrate. This product will be referred to as “slowly digestible starch” or SDS for the remainder of the report. This SDS is a blend of corn starch and tapioca flour, designed to deliver balanced energy and a reduced glycemic response when added to non-thermal applications. The aims of this study were to (1) characterize the in vitro digestibility profile of SDS; (2) confirm in vivo digestibility using a rooster model and (3) assess its glycemic response in a healthy population.

## 2. Materials and Methods

### 2.1. In Vitro Testing

The in vitro digestion method compared maltodextrin (Globe^®^ Plus 10 DE maltodextrin, Ingredion Incorporated, Bridgewater, NJ, USA), and SDS (SUSTRA™ 2434 slowly digestible carbohydrate, Ingredion Incorporated, Bridgewater, NJ, USA). A modified version of the Englyst method [8] was used to analyze the starches for RDS, SDS and RS contents. Three minor modifications were made to the assay: (1) a commercial source of amyloglucosidase (AMG) was used to replace the original AMG 400, which is no longer available; 1 mL AMG enzyme (Sigma A7095, from *Aspergillus niger*, ≥260 U/mL, St. Louis, MO, USA) was added to centrifuged 12 g pancreatin in solution; (2) Invertase (40 mg) (Sigma I4505, Grade VII from baker’s yeast, ≥300 U/mg) was added, replacing 4 mL of the original liquid enzyme (3000 EU/mL and (3) Guar gum (Sigma G4129) was prehydrated in 0.05 M HCl solution (0.5% *w*/*v*) with 0.5% pepsin before being added to the digestion tubes. Released glucose was determined using glucose oxidase/peroxidase (GOPOD) reagent. Slowly available glucose (SAG) was calculated using the following equation: SAG = (slowly digestible starch/glucose amount release at 120 min) × 100. The total dietary fiber of test starch was measured using the AOAC 991.43 method [15].

### 2.2. In Vivo Rooster Study

The aforementioned SDS and maltodextrin were evaluated in roosters. Nutrient digestibility is determined by measuring nitrogen-corrected true metabolizable energy (TME_n_) content of a starch and maltodextrin, using both conventional and cecectomized roosters, as previously described [16,17]. Briefly, two precision-fed rooster assays, utilizing conventional Single Comb White Leghorn roosters and cecectomized Single Comb White Leghorn roosters, were conducted. All animal housing, handling, and surgical procedures were approved by the University of Illinois Animal Care and Use Committee. Aliquots of the test carbohydrates were mixed with either 20 or 33 mL of water prior to dosing. After 26 h of feed withdrawal, 10 conventional roosters (5 roosters per treatment) and 10 cecectomized roosters (5 roosters per treatment) were tube-fed an average of 26.7 g (dry matter basis) of the test starches. Following crop intubation, excreta (urine and feces) were collected for 48 h on plastic trays placed under each individual cage. Excreta samples then were lyophilized, weighed, and ground prior to analysis. The two test carbohydrates were analyzed for dry matter (DM; 105 °C; AOAC, 2006; method 934.01) [18], and both the two test carbohydrates and the excreta samples were analyzed for N or crude protein (CP; TruMac^®^ N, LECO Corporation, St. Joseph, MI, USA; AOAC, 2006), and gross energy (GE) using a bomb calorimeter (Parr Instruments, Moline, IL, USA). The TME_n_ values, corrected for endogenous energy excretion based on previous data, were calculated using the following equation:

$$TME_{n}(\mathrm{kcal}/g)=\frac{EI_{fed}-\;(EE_{fed}+8.22\times N_{fed})+\left(EE_{fasted}+8.22\times N_{fasted}\right)}{FI}$$

where *EI_fed_* equals the gross energy intake of the test substrate consumed; *EE_fed_* equals the energy excreted by the fed birds; 8.22 is the correction factor for uric acid; *N_fed_* equals the g nitrogen retained by the fed birds; *EE_fasted_* equals the energy excreted by the fasted birds; *N_fasted_* equals the g nitrogen retained by the fasted birds; and *FI* equals the grams of dry test substrate consumed. The database with conventional and cecectomized birds indicates that the N-corrected endogenous energy excretion by fasted birds was 9.25 kcal.

### 2.3. Clinical Study

This study was conducted in accordance with the ethical principles outlined in the Declaration of Helsinki and the protocol was approved by the Western Institutional Review Board (Vancouver, BC, Canada). All subjects provided written informed consent prior to starting the study. The clinical study was conducted at GI Labs (Toronto, ON, Canada).

#### 2.3.1. Subject Screening

*Inclusion criteria*: Participants were healthy male or non-pregnant females, 18–75 years of age, with a body mass index (BMI) of ≥20 and ≤40 kg/m^2^. Participants were required to maintain their regular diet, supplement intake, physical activity and body weight throughout the study duration and refrain from smoking prior to each visit. On test days, subjects were not allowed to take any dietary supplements, until dismissal from the GI labs. Subjects were required to have normal fasting serum glucose (<7.0 mmol/L capillary corresponding to whole blood glucose <6.3 mmol/L), abstain from alcohol consumption and to avoid vigorous physical activity for 24 h prior to all test visits. Subjects had to have an understanding of the study procedures and be willing to provide informed consent to participate in the study and authorization to release relevant protected health information to the investigator.

*Exclusion criteria*: Subjects were excluded if they failed to meet inclusion criteria, had a history of chronic disease, such Type 1 or 2 Diabetes, cardiovascular disease, cancer, gastrointestinal disorders; used medications within four weeks of the screening, had surgery within 3 months of screening, had an intolerance or allergy to test ingredients, had extreme dietary habits, had drastic body weight changes >3.5 kg within four weeks of screening duration, had the presence of any symptoms of an active infection during screening or study visits, had a history of alcohol or substance abuse, or had an unwillingness or inability to comply with the experimental procedures and to follow GI Labs safety guidelines.

#### 2.3.2. Study Design and Subjects

**Figure 1:**
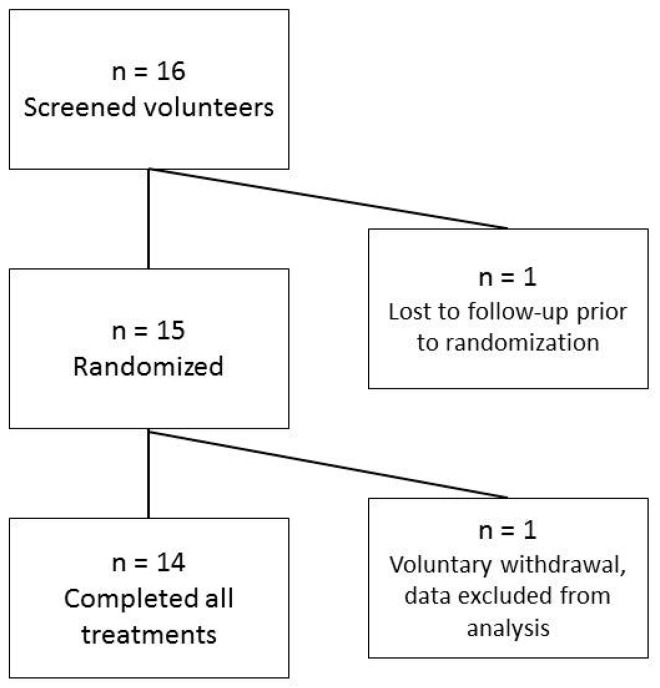
Subject flow through study.

The study was a randomized, double-blinded, placebo controlled, cross-over design, with 14 healthy adults (age 18–75 years, body mass index (BMI) ≥20.0 and <40.0 kg/m^2^). Eligible participants were studied on separate days over a period of 2 to 6 weeks. The interval between successive tests was no less than 48 h and no more than 2 weeks. Subjects completed six study visits in a random order, during which they consumed one of the following treatments: SDS bar, control bar, SDS pudding, control pudding, dextrose beverage 1 (50 g dextrose in 250 mL water) or dextrose beverage 2 (50 g dextrose in 250 mL water). The dextrose beverage was administered twice for glycemic index calculations. The study subject flow is shown in Figure 1. Descriptions of the study foods are provided in Section 2.3.3.

#### 2.3.3. Study Foods

*Cold-pressed bar:* The SDS bar and control bar were similar in appearance and were packaged in an opaque envelope with an alpha-numeric code for identification. Neither the study subjects nor the investigators knew the identity of the study foods. Both the SDS bar and control bar were matched for available carbohydrates (50 g). The SDS bar contained 24.9 g SDS (SUSTRA^TM^ 2434 slowly digestible carbohydrate, Ingredion Incorporated, Bridgewater, NJ, USA) and 18.9 g corn syrup (Globe^®^ Plus 63 DE maltodextrin, Ingredion Incorporated, Bridgewater, NJ, USA) while the control bar contained 22.1 g maltodextrin (Globe^®^ Plus 10 DE maltodextrin, Ingredion Incorporated, Bridgewater, NJ, USA) and 21.8 g corn syrup (Globe^®^ Plus 63 DE maltodextrin, Ingredion Incorporated, Bridgewater, NJ, USA). The portion sizes and nutrient composition of the study bars are shown in Table 1.

*Pudding:* The SDS pudding and control pudding were identical in appearance and were packaged in an opaque envelope with an alpha-numeric code for identification. Neither the study subjects nor the investigators knew the identity of the study foods. Both the SDS pudding and control pudding were matched for available carbohydrate (50 g). The test pudding contained 30.4 g SDS (SUSTRA^TM^ 2434 slowly digestible carbohydrate, Ingredion Incorporated, Bridgewater, NJ USA) and 10.9 g dextrose (CERELOSE^®^ dextrose, Ingredion Incorporated, Bridgewater, NJ, USA), and the control pudding contained 39.6 g dextrose (CERELOSE^®^ dextrose, Ingredion Incorporated, Bridgewater, NJ, USA). The remainder of available carbohydrate in both puddings was provided by whole (full-fat) milk. The puddings were prepared by mixing an entire 300 g package of powder with 840 g whole milk, whisking for 2 min and refrigerating overnight. The portion sizes and nutrient composition of the study puddings are shown in Table 1.

#### 2.3.4. Study Visit Procedures

Participants were asked to maintain stable dietary and activity habits throughout the study. Prior to each study visit, participants refrained from drinking alcohol and from unusual levels of food intake or physical activity for 24 h. On each test occasion, subjects arrived at the clinical site after fasting for 10 to 12 h. Two fasting blood samples for glucose analysis (2–3 drops into a fluoro-oxalate tube) were obtained by fingerprick, 5 min apart and after the second sample, the subject started to consume a test meal. Each test meal was served with a drink of 1 or 2 cups of coffee or tea with 30 mL of 2% milk if desired, or water. At the first visit, each subject selected the type and volume of drink desired and the same type and volume of drink was consumed on subsequent visits. Subjects consumed the entire test meal within 10 min. At the first bite, a timer was started and additional blood samples for glucose analysis (2–3 drops into a fluoro-oxalate tube) were taken at 15, 30, 45, 60, 90, 120, 150, 180, 210 and 240 min after starting to eat. Blood samples were obtained from hands warmed with an electric heating pad for 3–5 min prior to each sample.

### 2.4. Biochemical Analysis

After blood collection the tubes containing blood for glucose analysis were rotated to mix the blood with an anti-coagulant and then placed in a refrigerator until the last blood sample in the set had been collected. After all tubes were collected from one subject, the tubes were stored in a −20 °C freezer until analysis. Analysis was performed within 3 days of the study visit, using a YSI model 2300 STAT analyzer (Yellow Springs, OH, USA).

### 2.5. Data Analysis and Statistics

#### 2.5.1. Sample Size Calculation for Clinical Study and Randomization Method

The main effect of treatment was expected to have a SD of 23%/sqrt (2) because each subject tested each treatment in 2 types of food (bar and pudding). We wished to be able to detect a difference in glycemic response of 20%; *n* = 14 subjects provided 80% of the power required to detect a difference of 19%.

The study was conducted in two blocks of three treatments; the two blocks contained either the bar or the pudding and within each block the tests consisted of one dextrose test and the control and test food. The six possible orders of three treatments were listed twice, one for each block. The six orders in each block were randomized using the Rand () function on an Excel spreadsheet. The order of the blocks was randomized in the same way and the process was repeated three times.

The order of testing was assigned to the 14 subjects in the order they attended for the first visit; the extra orders were included in case of drop-outs which needed to be replaced.

#### 2.5.2. Data Analysis

Glycemic index values were calculated based on previously published methods [19]. The net incremental area under the curve was calculated using the trapezoidal rule. Values below the baseline were treated as negative values.

#### 2.5.3. Statistical Analysis

Paired t-tests were conducted on blood glucose values at individual time points, net incremental area-under-curve (iAUC), and glycemic index using GraphPad Prism 7 (v 7.03, GraphPad Software, Inc., La Jolla, CA, USA). *p* values < 0.05 were deemed statistically significant.

## 3. Results

### 3.1. In Vitro Testing

**Figure 2:**
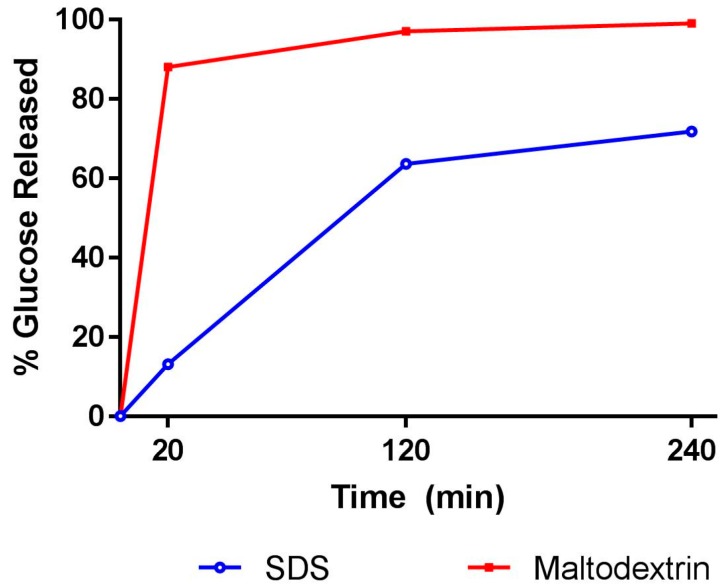
In Vitro digestibility profile of slowly digestible starch (SDS) and maltodextrin.

The digestibility profile of SDS and maltodextrin (control) was determined by measuring glucose release, as shown in Figure 2. Glucose released in the first 20 min reflects RDS, while the SDS fraction was obtained by subtracting the amount of glucose hydrolyzed at 120 min with glucose released at 20 min (120 min–20 min). Glucose release beyond 120 min reflects the resistant starch fraction. The tested SDS showed a 13% rapidly digestible fraction and 51% was slowly digestible, while in the control, 88% was rapidly digestible and 9% was slowly digestible. At the end of two hours digestion, slowly available glucose (SAG) was higher for SDS (79.4%), as compared to the control (9.3%). Total dietary fiber of SDS measured by AOAC 991.43 method was 10%, as-is.

### 3.2. In Vivo Rooster Study

Roosters provide a robust model to determine digestibility and/or fermentability of an ingredient. The true metabolizable energy (TMEn) for both SDS and maltodextrin (control) was high, indicating their complete digestibility (Table 2). However, the energy value of SDS was slightly lower than the maltodextrin in roosters, suggesting that around 6% is non-digestible, aligning with the fiber fraction.

### 3.3. Clinical Study

Study demographics are shown in Table 3. All participants were healthy.

The inclusion of SDS in a cold-pressed bar and instant pudding yielded a significantly lower glycemic index compared to the control (Table 4).

**Figure 3:**
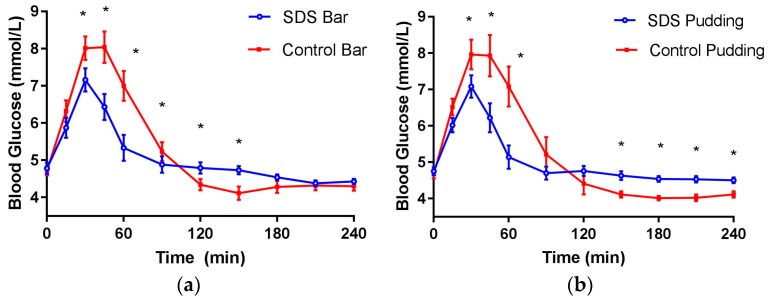
Post-prandial glycemic response of SDS in healthy adults (**a**) cold-pressed bars (**b**) instant pudding. Data are mean ± standard error mean (SEM); * indicates treatments were significantly different at specific time points in the paired *t*-test (*p* < 0.05).

SDS inclusion in the cold-pressed bar yielded a significantly different mean blood glucose concentration (Figure 3a) and a 50% lower net iAUC (0–2 h) (Table 4), compared to control bars. The SDS bar resulted in significantly lower blood glucose values at 30, 45, 60 and 90 min, and significantly higher blood glucose values at 120 and 150 min, compared to the control (Figure 3a). Similarly, SDS inclusion in pudding yielded significantly different mean blood glucose concentrations (Figure 3b) and a 56% lower net iAUC (0–2 h), compared to the control pudding. The SDS pudding resulted in significantly lower blood glucose values at 30, 45, and 60 min, and significantly higher blood glucose values at 150, 180, 210, and 240 min, compared to the control (Figure 3b). The blood glucose concentrations in response to SDS in the bar and pudding after two hours remained above the baseline for an average of 15 min longer compared to the control foods, and the drop below baseline was less substantial for SDS incorporated foods.

## 4. Discussion

Global health concerns, such as diabetes, can be addressed with lifestyle changes, including dietary modifications by replacing rapidly digestible carbohydrates with slowly digestible carbohydrates. Natural SDCs, such as whole grains and pulses, comprise slowly digestible starch and resistant starch/fiber fractions. Dietary fiber has profound health benefits, but can lead to gastrointestinal discomfort. Thus, there is a heightened interest in ingredients with high amounts of SDS and low RDS content, in order to deliver sustained glucose energy.

Carbohydrate quality or prolonged digestion is an emerging area and studies to evaluate the effect of SDCs are currently relying on glycemic response and glycemic index measurements to define “sustained energy” [20]. However, “sustained energy” may have multiple consumer interpretations [21]. Not all carbohydrates are the same, with respect to digestion and absorption. Some carbohydrates, such as maltodextrin, are rapidly digested and absorbed, which results in a rapid blood glucose spike and crash. This places greater demands on the pancreas and the liver to manage the carbohydrate load. In contrast, SDS results in a steadier blood glucose rise and a slower blood glucose drop [13].

This is the first study to characterize a SDS by adopting a translational approach, which involved in vitro screening of the ingredient and in vivo validation of slow but near complete digestibility and prolonged glucose release. In the in vitro assay, SDS had an over 50% slowly digestible fraction and showed a steady glucose release over 120 min of digestion which simulates digestion in the small intestine. SDS had low fiber and RDS fractions. Carbohydrate digestibility in humans is commonly determined in ileostomates [22]. Alternately, the rooster model provides a quick and reliable tool to measure true metabolizable energy of ingredients [23]. SDS in roosters showed high digestibility similar to control, but the energy value was slightly lower than the control, indicating slower glucose release. In Vitro and rat studies on hydrogenated isomaltulose also showed slower hydrolysis and resulted in lower glycemic response [24].

A recently published meta-analysis [25], with three clinical trials, including 79 subjects, showed a direct association of SDS content on glucose metabolism. A high-SDS containing cereal had a 15-fold higher chance of having a low rate of appearance of exogenous glucose (RaE). In the current study, SDS formulated in cold-pressed bars and pudding showed a significantly lower glucose response, iAUC and glycemic index, compared to the control foods with high RDS. The low glycemic response may be attributed to the high content of SDS and slowly available glucose, beyond 2 h of digestion. A similar study with two native granular uncooked starches, which differed in digestion rate, was evaluated for glycemic and insulin responses in healthy and diabetic populations [26]. Slowly hydrolyzed starch significantly lowered glycemic and insulin responses over 6 h and reduced 3-hydroxybutyrate, an indicator for sustained use of glucose as oxidizable fuel.

Another study with starches differing in hydrolysis rates, indicated a relation between starch in vitro digestibility and glycemic response in healthy men [27]. Subjects consumed five soups containing 50 g maltodextrin (SDS = 1 g), whole-grain (SDS = 4 g), high-amylose starch (SDS = 6 g), regular cornstarch (SDS = 16 g) or no added starch at 1-week intervals. Ad libitum food intake was measured at 30 min or 120 min, which were the estimated times of digestion of a rapidly digestible starch (RDS) and slowly digestible starch, respectively. Blood glucose concentrations and appetite were measured pre- and post-meal. Food intake was significantly reduced by maltodextrin at 30 min and by whole-grain, high-amylose starch and corn starch at 120 min. Blood glucose AUC was significantly lower for all starches at 30 min, while at 120 min blood glucose AUC was significantly reduced for corn starch. This study showed that ingredients with high SDS contents impact digestibility, glycemic response and satiety.

Glycemic responses of other sugar-based SDCs, such as isomaltulose and sucromalt, were tested in low GI formulated meal replacement drinks in diabetics [28]. Four enteral formulas were tested. Two drinks had SDCs, which were compared to a standard formula and high-fat drink. SDC-enriched and high-fat beverages significantly attenuated blood glucose and blood insulin concentrations, while the high-fat formula had a greater reduction effect on triglycerides. However, the SDC content of these slowly hydrolyzed carbohydrates has not been reported.

The current study has few limitations. The modified in vitro method is a screening tool to characterize digestibility profile and is less dynamic in terms of measuring interactions with other nutrients and absorption uptake and does not completely reflect physiological responses. The human ileostomate model, which has been utilized to measure ingredient digestibility in some studies, was replaced with the cost-effective rooster model. Other limitations included lack of insulin response measurements, rate of glucose appearance and indicators of carbohydrate and fat oxidation.

## 5. Conclusions

The SUSTRA™ 2434 slowly digestible carbohydrate is a blend of tapioca flour and corn starch, with the potential to provide balanced energy. The new SDS has been shown to be highly digestible in vivo and yielded a lower GI, compared to a rapidly digestible control. Incorporating SDS into a cold-pressed bar and instant pudding resulted in a lower glucose response in the first 60–90 min and a higher glucose response at two hours and beyond, suggesting steadily available energy. The reduced blood glucose response to SDS is due to the presence of a significant slowly digestible starch fraction and low rapidly digestible and resistant fractions, as documented in the in vitro study. The balanced energy from the SUSTRA™ 2434 slowly digestible carbohydrate is a solution to improve carbohydrate quality in non-thermal applications.

## Figures and Tables

**Table 1:** Nutrient composition of cold-pressed bars and instant pudding. SDS: slowly digestible starch.

Nutrient Content (g)	Bar SDS	Bar Control	Pudding SDS	Pudding Control
Serving size	72	70	200	200
Total carbohydratess	52.9	52.8	52.6	52.4
Available carbohydratess	50.1	51.0	50.0	50.7
Sugars	19.0	20.7	17.0	43.4
Dietary fiber	2.8	1.9	2.6	1.7
Protein	4.8	4.4	5.8	5.7
Fat	4.4	4.2	5.6	5.6

**Table 2:** True metabolizable energy (TMEn) evaluation of slowly digestible starch (SDS) in roosters.

Ingredients	Gross Energy ^1^	TMEn	Digested (%)
SDS	3.986	3.756	94.2
Maltodextrin	4.008	4.014	100

^1^ Bomb calorimetry, all values are kcal/g, based on dry-basis.

**Table 3:** Subjects demographics.

Mean ± Standard Deviation (SD)	Participants (*n* = 14)
Age (years)	38.3 ± 13.3
Gender (male/female)	10/4
Weight (kg)	78.6 ± 10.4
Body mass index (kg/m^2^)	26.8 ± 2.7
Fasting blood glucose (mg/dL)	82.5 ± 8.8

**Table 4:** Glycemic index and net incremental area-under-curve (iAUC) for SDS and control.

	Bars			Puddings		
Ingredient	SDS	Control	*p*-Value	SDS	Control	*p*-Value
Glycemic Index ^1,^*	49.9 ± 4.6	93.0 ± 8.1	<0.0001	45.9 ± 4.5	92.6 ± 9.2	<0.0001
Net iAUC (0–2 h) *	91.8 ± 16.9	183.4 ± 23.1	0.0001	83.7± 14.8	191.1 ± 33.5	0.014

^1^ Glycemic Index (GI) Scale: Low GI: ≤55; Medium GI: 56–69; High GI: ≥70. * Values are presented as mean ± standard error mean (SEM), control vs SDS are statistically different in the *t*-test if *p* < 0.05.
